# Molecular Detection of Vertebrates in Stream Water: A Demonstration Using Rocky Mountain Tailed Frogs and Idaho Giant Salamanders

**DOI:** 10.1371/journal.pone.0022746

**Published:** 2011-07-26

**Authors:** Caren S. Goldberg, David S. Pilliod, Robert S. Arkle, Lisette P. Waits

**Affiliations:** 1 Fish and Wildlife Resources, University of Idaho, Moscow, Idaho, United States of America; 2 United States Geological Survey, Forest and Rangeland Ecosystem Science Center, Boise, Idaho, United States of America; Smithsonian's National Zoological Park, United States of America

## Abstract

Stream ecosystems harbor many secretive and imperiled species, and studies of vertebrates in these systems face the challenges of relatively low detection rates and high costs. Environmental DNA (eDNA) has recently been confirmed as a sensitive and efficient tool for documenting aquatic vertebrates in wetlands and in a large river and canal system. However, it was unclear whether this tool could be used to detect low-density vertebrates in fast-moving streams where shed cells may travel rapidly away from their source. To evaluate the potential utility of eDNA techniques in stream systems, we designed targeted primers to amplify a short, species-specific DNA fragment for two secretive stream amphibian species in the northwestern region of the United States (Rocky Mountain tailed frogs, *Ascaphus montanus*, and Idaho giant salamanders, *Dicamptodon aterrimus*). We tested three DNA extraction and five PCR protocols to determine whether we could detect eDNA of these species in filtered water samples from five streams with varying densities of these species in central Idaho, USA. We successfully amplified and sequenced the targeted DNA regions for both species from stream water filter samples. We detected Idaho giant salamanders in all samples and Rocky Mountain tailed frogs in four of five streams and found some indication that these species are more difficult to detect using eDNA in early spring than in early fall. While the sensitivity of this method across taxa remains to be determined, the use of eDNA could revolutionize surveys for rare and invasive stream species. With this study, the utility of eDNA techniques for detecting aquatic vertebrates has been demonstrated across the majority of freshwater systems, setting the stage for an innovative transformation in approaches for aquatic research.

## Introduction

Freshwater systems are hotspots for both biodiversity and species endangerment [1], with freshwater fauna experiencing 123 documented extinctions in the 20^th^ century [2]. Growing demand for water resources indicates that threats to freshwater species will further increase over the next century [3]. Stream species are particularly vulnerable to cumulative changes in land cover [4], [5], climate [6], and biotic and abiotic inputs [7], [8]. Migratory stream salmonids (e.g. bull trout, *Salvelinus confluentus*, and Chinook salmon, *Oncorhynchus tshawytscha*) are among the most imperiled North American fishes [9] and the most catastrophic documented amphibian population declines have been in streams [10]. Additionally, streams are increasingly being invaded, at great ecological and economic costs, by exotic species, including crayfish, aquatic mussels, and gastropods [11], [12].

Investigations into the distribution and ecology of stream species are often hindered by the challenges of working in these systems. Stream species are difficult to inventory due to the complexity of topography and vegetation in streambeds and riparian areas, water turbidity and flow rate, low densities of individuals, cryptic coloration, and the use of microhabitats. Due to these and other factors, surveys for native and exotic species in streams can be expensive and inaccurate [13], [14]. For example, a major challenge in amphibian decline research is that amphibians can be difficult to detect, especially in streams [15]. Electrofishing techniques have high success for detection of aquatic vertebrates in many cases [16], but can be time consuming and difficult to apply in streams, and may cause injury to target and non-target species [17].

Researchers have been using DNA from feces, urine, hair, feathers, shed skin, and eggshells to detect terrestrial vertebrate species for the past decade [18], and detection of microbial species using environmental DNA (eDNA) found in soil and seawater is revolutionizing species inventories [19] and enabling efficient disease detection [20]. Recently, the reliable detection of aquatic vertebrate species using eDNA in water was confirmed in wetlands [21] and in a large river and canal system [22]. Using eDNA to detect rare and secretive species in streams could increase accuracy and decrease costs of surveys, increase the number of sites sampled per unit effort, refine distribution and extinction records, and provide early detection of invasive species in these systems, without any risk to the species. However, the fast flow of streams may move shed cells away from their source at a rate prohibitive to eDNA collection. To evaluate the potential for using eDNA to survey for stream species, we collected water samples from five small headwater streams in two seasons and tested them for DNA of two amphibian species (Rocky Mountain tailed frogs, *Ascaphus montanus*, and Idaho giant salamanders, *Dicamptodon aterrimus*) known to be present at the sites. To achieve this, we designed species-specific primers and tested multiple DNA extraction and PCR protocols designed to amplify low quality DNA templates.

## Methods

In the first phase of the project, we collected one 10-L and two 5-L water samples from a headwater stream (Table 1) with known presence of two species of amphibians (Rocky Mountain tailed frogs and Idaho giant salamanders) in late September of 2010 using a flow-through filter with a peristaltic pump and 0.45 µm cellulose nitrate filter paper (State of Idaho Wildlife Collection Permit #030716 and Payette National Forest Research Permit #0105). Each filter was preserved in 95% ethanol in a separate 2 mL tube. We estimated the larval density of both species at this site using standard kick-sampling methodology [23] in July and August 2010. Density survey and water sample collections were made during base flow, measured as 0.23 m^3^ s^−1^, in the study stream.

**Table 1:** Sampling sites, dates of sampling, PCR success for each species, and densities of Idaho giant salamanders (*Dicamptodon aterrimus;* DIAT) and Rocky Mountain tailed frogs (*Ascaphus montanus*; ASMO) where stream filter samples were taken, estimated using field methods in summer 2010.

Site	Latitude	Longitude	Date sampled	DIAT per m^2^	DIAT PCR success (%)	ASMO per m^2^	ASMO PCR success (%)
**Phase 1**							
Nasty Creek	44.877	−115.696	25Sept10	0.032	100	0.228	100
**Phase 2**							
Camp Creek	44.890	−115.706	27Mar11	0.036	100	0.097	16.7
Deadman Creek	44.966	−115.663	27Mar11	0.011	100	0.149	0
Goat Creek	44.759	−115.684	27Mar11	0.029	100	0.052	33.3
Nasty Creek	44.877	−115.696	03Apr11	0.032	100	0.228	33.3
Reegan Creek	44.949	−115.587	27Mar11	0.011	100	0.337	16.7

We designed a set of species-specific primers for each species targeting a small region of the mitochondrial DNA (mtDNA) cytochrome *b* gene (obtained from GenBank) [24], [25] (Table 2). The distribution of these two species is disjunct from their congeners along the Pacific coast to the west; therefore, we designed primers to be species-specific within our system (the northern Rocky Mountains region) but also to detect the congeners of each species for wider geographic applicability. Target fragment length was 78 base pairs for *Dicamptodon* and 85 base pairs for *Ascaphus*. This test was designed to amplify previously-published sequences characteristic of these species; no new sequence data was generated that had not already been published. All extractions and PCR set-up were done in a room dedicated to low-quantity DNA sources; no DNA from amphibians had previously been handled in this room.

**Table 2:** Primer sequences for species-specific amplification of short fragments of cytochrome *b*.

Species	Primer name	Primer sequence
Rocky Mountain tailed frog(*Ascaphus montanus*)	ASMO F	CGT CAA CTA TGG CTG GCT AA
	ASMO R	TCG GCC AAT GTG AAG ATA AA
Idaho giant salamander(*Dicamptodon aterrimus*)	Dicamp F	TCT GCA TCT TYC TAC ATA TYG GAC
	Dicamp R	ATC ACY CCG ACK TTT CAG GT

In this first phase, we tested two DNA extraction and three PCR protocols for the detection of Rocky Mountain tailed frogs and Idaho giant salamanders using eDNA from these filter samples. First, we removed the filters from the ethanol and air-dried them overnight. We then divided each filter in half and extracted each half with either the DNeasy Tissue and Blood Kit (Qiagen, Inc.) or the UltraClean® Soil DNA isolation kit (MoBio Laboratories, Inc.). We then attempted to amplify DNA from each sample using a standard PCR protocol (PCR Protocol 1; Table S1). All reactions included a negative extraction control, negative PCR control, and positive controls for each of the target species. We ran PCR products on 3% agarose gels to determine success. When this first protocol produced no PCR products, we reran the reactions with a combination of each DNA sample and a positive control in each tube to determine whether PCR inhibitors were preventing amplification. For samples extracted using the Qiagen DNeasy kit, we also tested three PCR protocols (PCR Protocols 1, 2, and 3; Table S1) with a dilution series of each sample (1X, 0.1X, 0.01X, and 0.001X).

We sequenced products of the most successful combination of protocols using the BigDye system on a 3130xl capillary sequencer (Applied Biosystems). To streamline the assay for large-scale application, primers were labeled with fluorescent dyes and a PCR multiplex was created using primer sets for both species with PCR Protocol 3. We tested additional negative controls of DNA from sympatric amphibian species (*Ambystoma macrodactylum, Bufo boreas, Pseudacris sierra, Rana luteiventris*) with this multiplex, independently (1 reaction/species; approximately 5 – 100 ng DNA/reaction) and together with DNA from the target species, to verify the specificity of our diagnostic test.

In the second phase of the project, we collected a 5-L water filter sample from each of five headwater streams known to contain populations of Rocky Mountain tailed frogs and Idaho giant salamanders, including the original stream (Table 1). Streams were sampled in late March and early April 2011 using the same field collection techniques as above; density estimates for these streams were obtained July and August 2010 (State of Idaho Wildlife Collection Permit #030716 and Payette National Forest Research Permit #0105). We used the DNeasy extraction method and PCR Protocol 3 (Table S1) for one half of each filter and a modified extraction, with the addition of the use of a QIAshredder (Qiagen, Inc.) after overnight digestion with Proteinase K, and PCR Protocol 4 (Table S1) for the other half of each filter. We also tested the Qiagen Multiplex Plus PCR kit with this modified protocol (PCR Protocol 5). These samples were only run at full concentration. We tested whether field-estimated densities predicted PCR success for these samples using simple linear regression in R 2.13.0 [26].

## Results

**Figure 1:**
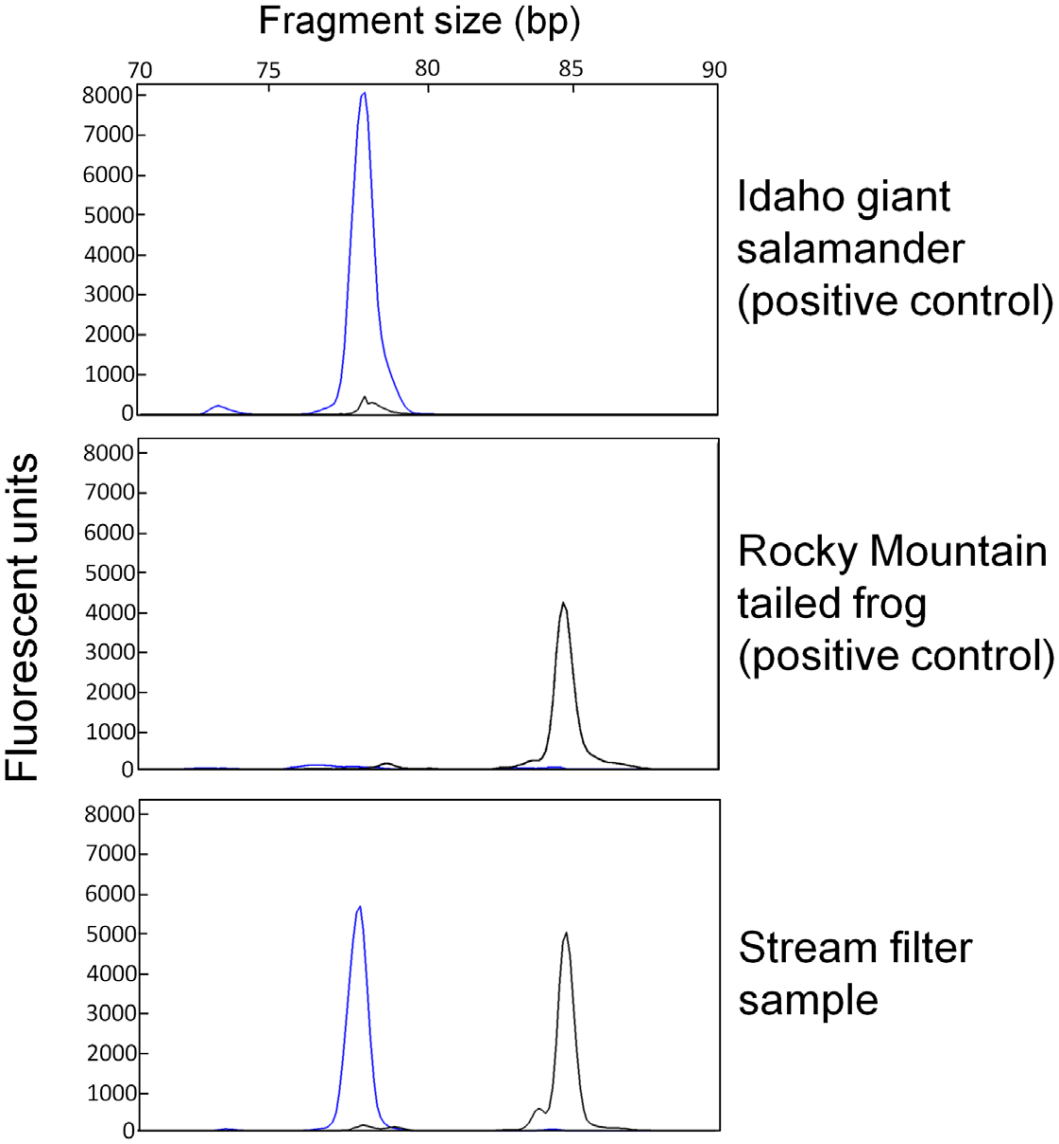
Electropherograms of species-specific PCR amplification of DNA in positive controls and stream water. The blue peak indicates the species-specific fragment for Idaho giant salamanders (*Dicamptodon aterrimus*) and the black peak indicates the species-specific fragment for tailed frogs (*Ascaphus montanus*). All reactions were diluted to produce these images.

In the first phase of the project, we recovered the targeted DNA sequence from both species from all stream water filter samples only when using the DNeasy extraction method and PCR Protocol 3. For two of the three samples (one 5-L, one 10-L) the correct fragment was also detectable at 0.1X DNA concentration for Rocky Mountain tailed frogs and down to 0.001X DNA concentration for Idaho giant salamanders. Tests for inhibition with PCR Protocol 1 showed that samples from the DNeasy extraction method were inhibited (but the extraction negative control was not), while samples from the MoBio extraction were not inhibited, indicating the lack of target species DNA in the results of these extractions. PCR multiplexing with fluorescently-tagged primers provided clear and efficient detection of amplified fragments (Fig. 1), with all samples and positive controls at >8000 fluorescent units, even when DNA from the target species was mixed with DNA from non-target species. None of the negative controls, including DNA from four sympatric amphibian species, tested positive.

In the second phase of the project, where samples were collected in the early spring, we detected Idaho giant salamanders in all filter samples using PCR Protocol 3 but did not detect Rocky Mountain tailed frogs. Amplifications for Idaho giant salamanders were weak (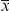  =  203, 95% C.I. 25 – 382 fluorescent units) compared with samples collected in early fall. With the addition of the QIAshredder kit and using PCR protocol 4, we detected both species in all but one of the streams, with strong signal for Idaho giant salamanders in all reactions (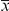  =  5962, 95% C.I. 4555 – 7369 fluorescent units) and detection probability across 6 PCR replicates for Rocky Mountain tailed frogs ranging from 0 to 33% (Table 1; for successful amplifications, 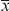  =  7636, 95% C.I. 6176 – 9097 fluorescent units). Substitution of the Qiagen Multiplex Plus PCR kit for the Qiagen Multiplex PCR kit in PCR Protocol 5 did not improve performance (Table S1). There was no evidence that the probability of detection of Rocky Mountain tailed frogs in a PCR replicate was related to field-estimated density from the previous summer (PCR Protocol 4; *F*_1,3_ = 0.036, *P* = 0.86).

## Discussion

Using filter samples taken from stream water, we developed an efficient protocol for detecting targeted DNA sequences for two secretive amphibian species, demonstrating that the recovery of amphibian DNA from stream water is possible even when amphibian populations are at low densities. The rapid field collection protocol, relatively simple field equipment and low cost (supply cost per sample with 6 PCR replicates  =  $10.11) make this technique widely applicable to broad-scale inventory and monitoring efforts. The probability of detection of eDNA across densities likely varies with species, stream size, and discharge rate, and by season, as suggested by this study. However, the potential impact of this technique for inventorying species in stream systems is far-reaching, including detection of rare or imperiled vertebrates.

We found that only the DNeasy Blood & Tissue kit with the Qiagen Multiplex PCR kit detected eDNA for both species in water filter samples. We did not successfully extract DNA from the filter samples using the UltraClean® Soil DNA isolation kit (MoBio Laboratories, Inc.); possibly the PowerWater DNA Isolation kit (MoBio Laboratories, Inc.) used to detect eDNA of Asian carp [22] would have yielded better results. Our results indicate that using the Qiagen Multiplex PCR kit improves species detection in water filter samples over a protocol using Amplitaq Gold DNA polymerase and BSA; this latter combination was used to establish that the detection of aquatic vertebrates using eDNA in water samples was possible [21].

Although we only sampled one stream in the first phase of our project, our results suggest that detection of Rocky Mountain tailed frogs and Idaho giant salamanders using eDNA may be more difficult when samples are taken in early spring rather than early fall. This could be due to decreased metabolism during cold weather or changes in behavior of the target species, such as moving into the hyporheic zone. For Idaho giant salamanders, we were able to compensate for this by modifying protocols, but for Rocky Mountain tailed frogs, detectability was still relatively low in early spring samples. This difference between species may be due to species-specific seasonal changes in density; while streams in the spring are likely to have one fewer Rocky Mountain tailed frog tadpole cohort than in the early fall due to timing of metamorphosis [27], the difference in overall population density is likely less extreme for Idaho giant salamanders because they are commonly neotonic [28]. This result demonstrates that sampling design for eDNA needs to be informed by the ecology of target species to maximize detection probabilities.

Our approach was to design species-specific primers to detect species of interest; these kinds of targeted primers can be multiplexed to test for many species in a single PCR reaction. However, when the species list is large or inventory for unknown species is the goal of sampling, universal primers and next-generation sequencing techniques could be applied [19]. Using these tools, researchers would sample a stream, river, or wetland, use primers that work across taxa to amplify DNA from this sample, and compare the sequences to those available in a reference library [29]. If sequences are recovered that do not match any in the library, sequences that are closest matches could be used to determine the probable taxonomic group of the unknown species and additional field surveys could be conducted to attempt to locate the species. Next-generation sequencing is currently prohibitively expensive for large survey efforts, but costs will likely be greatly reduced in the near future as the technology improves [30].

The success of eDNA for detecting vertebrates efficiently across freshwater systems indicates that this new tool has the potential to revolutionize surveys for aquatic species with the techniques currently available. The ability to survey for species across taxa with a single water sample would greatly enhance data availability for aquatic species and benefit resource managers and many fields of research, including community ecology, biogeography, evolutionary biology, conservation biology, and invasion biology. eDNA techniques could be used to form cost-efficient multi-species inventory and monitoring programs for sensitive species, in combination with occupancy models [31] to estimate probabilities of detection. With next-generation sequencing, DNA sequences of a community of aquatic vertebrates could be analyzed simultaneously, exponentially increasing the data available for analysis without disturbing sensitive species. Other applications include early detection of invasive species [21], [22], determining whether invasive species have been successfully removed through management actions, detecting rare individuals surviving after catastrophic population declines, and discovering new species in rapid bioassessement surveys. Sensitivity of these techniques to density of individuals and covariates of detection probability such as water temperature and discharge will need to be determined for study systems individually; however, this technique shows great potential for increasing our knowledge of aquatic systems.

## Supporting Information

Table S1 PCR protocols and results for amplifying DNA of Rocky Mountain tailed frogs (*Ascaphus montanus*) and Idaho giant salamanders (*Dicamptodon aterrimus*) from stream water.
(DOCX)
