# Supplementary material for: Molecular Detection of Vertebrates in Stream Water: A Demonstration Using Rocky Mountain Tailed Frogs and Idaho Giant Salamanders
Source: PLoS One. 2011 Jul 26;6(7):e22746. doi: 10.1371/journal.pone.0022746 (PMC3144250; doi:10.1371/journal.pone.0022746)
Supplement: Table S1 — PCR protocols and results for amplifying DNA of Rocky Mountain tailed frogs (Ascaphus montanus) and Idaho giant salamanders (Dicamptodon aterrimus) from stream water. (DOCX) [file pone.0022746.s001.docx]

Table S1. PCR protocols and results for amplifying DNA of Rocky Mountain tailed frogs (*Ascaphus montanus*) and Idaho giant salamanders (*Dicamptodon aterrimus*) from stream water.

| Extraction protocol | PCR protocol | Results |
| --- | --- | --- |
| *Phase 1 (early fall), Nasty Creek only, one 10-L and two 5-L samples* | | |
| MoBio Extraction: UltraClean® Soil DNA isolation kit (MoBio Laboratories, Inc.) | PCR protocol 1: 1 µl DNA sample, 1X AmpliTaq Gold PCR Buffer (Applied Biosystems), 3.0 mM MgCl_2_, 0.4 mM of each dNTP, 0.2 µM of ASMO primers or 0.3µM of Dicamp primers (Table 2), 0.4 units of AmpliTaq Gold DNA polymerase in a 10 µl reaction. Initial incubation at 94ºC for 15 minutes followed by 50 cycles of 94ºC for 1 minute, a touchdown step from 58-50ºC that decreased by 0.5ºC per cycle until the lower temperature was reached for 30 seconds, and 72ºC for 30 seconds, finishing at 72ºC for 5 minutes. | No product, not inhibited |
| DNeasy extraction: DNeasy Tissue and Blood Kit (Qiagen, Inc.) | PCR protocol 1 | Inhibited |
| DNeasy extraction | PCR Protocol 2: PCR Protocol 1 with addition of 0.75 mg/ml bovine serum albumin (BSA) | Both species detected in 10L sample only. |
| DNeasy extraction | PCR Protocol 3: 1X Qiagen Multiplex PCR Master Mix (Qiagen, Inc.), 0.5X Q solution, 0.2 µM of ASMO primers and/or 0.3 µM of Dicamp primers, and 1 µl extracted DNA in a 7 µl reaction. Initial incubation at 95ºC for 15 minutes followed by 50 cycles of 94ºC for 30 seconds, a touchdown step from 58-50ºC that decreased by 0.5ºC per cycle until the lower temperature was reached for 90 seconds, and 72ºC for 60 seconds, finishing at 60ºC for 30 minutes. | Both species detected in all samples. |
| *Phase 2 (early spring), 5 streams sampled, 5L each.* | | |
| DNeasy extraction | PCR Protocol 3 (multiplex) | Weak amplification for Idaho giant salamanders, no detection of Rocky Mountain tailed frogs. |
| DNeasy extraction, QIAshredder (Qiagen, Inc.) | PCR Protocol 4: PCR Protocol 3 (multiplex) without Q solution and with 55 total cycles | Rocky Mountain tailed frogs detected in 4/5 streams, Idaho giant salamanders in 5/5 streams. |
| DNeasy extraction, QIAshredder (Qiagen, Inc.) | PCR Protocol 5: PCR Protocol 4 with Qiagen Multiplex Plus PCR kit. | Rocky Mountain tailed frogs detected in 2/5 streams, Idaho giant salamanders in 4/5 streams. |
